# Obsessive-compulsive disorder reinforcement during the COVID-19 pandemic

**DOI:** 10.47626/2237-6089-2020-0054

**Published:** 2021-01-15

**Authors:** Felipe Ornell, Daniela Tusi Braga, Daniela Vicente Bavaresco, Ingrid Davila Francke, Juliana Nichterwitz Scherer, Lisia von Diemen, Felix Henrique Paim Kessler

**Affiliations:** 1 Centro de Pesquisa em Álcool e DrogasHospital de Clínicas de Porto AlegreUniversidade Federal do Rio Grande do SulPorto AlegreRSBrazil Centro de Pesquisa em Álcool e Drogas, Hospital de Clínicas de Porto Alegre (HCPA), Universidade Federal do Rio Grande do Sul (UFRGS), Porto Alegre, RS, Brazil.; 2 Programa de Pós-Graduação em Psiquiatria e Ciências do ComportamentoUFRGSPorto AlegreRSBrazil Programa de Pós-Graduação em Psiquiatria e Ciências do Comportamento, UFRGS, Porto Alegre, RS, Brazil.; 3 Laboratório de Psiquiatria MolecularPrograma de Transtorno BipolarHCPAPorto AlegreRSBrazil Laboratório de Psiquiatria Molecular e Programa de Transtorno Bipolar, HCPA, Porto Alegre, RS, Brazil.; 4 Universidade do Extremo Sul CatarinenseCriciúmaSCBrazil Universidade do Extremo Sul Catarinense (UNESC), Criciúma, SC, Brazil.; 5 Universidade Luterana do BrasilGuaíbaRSBrazil Universidade Luterana do Brasil (ULBRA), Guaíba, RS, Brazil.; 6 Universidade do Vale do Rio dos SinosSão LeopoldoRSBrazil Universidade do Vale do Rio dos Sinos (UNISINOS), São Leopoldo, RS, Brazil.

**Keywords:** COVID-19, pandemic, mental health, obsessive compulsive disorder

## Abstract

The COVID-19 pandemic is unquestionably impacting on the mental health of the population worldwide. Fear of contamination can both increase levels of stress in healthy individuals and intensify psychiatric symptoms in patients with pre-existing conditions, especially obsessive-compulsive disorder (OCD). During the COVID-19 pandemic, the imminent risk of contamination creates a logical need for self-surveillance and hygiene habits. However, this kind of information can have drastic implications for subjects with OCD, since cognitive distortions and compensatory strategies (cleansing rituals) are no longer irrational or oversized – rather, these ideas become legitimate and socially accepted, generating plausible validation for the intensification of compulsive cleaning rituals. Patients who presented remission of OCD symptoms would be more likely to have a relapse, and subclinical patients may scale up and ultimately be diagnosed with OCD due to the reinforcement of their habits, emotions and thoughts.

The COVID-19 pandemic is unquestionably impacting on the mental health of the population.^1,2^ Both fear of contamination and the socioeconomic effects of the pandemic can increase levels of stress in healthy individuals and intensify psychiatric symptoms in patients with pre-existing disorders.^3,4^ It is important to note that a disproportionate level of anxiety, accompanied or not by harmful behaviors, is an essential component of the development of some psychiatric disorders^5,6^ especially obsessive-compulsive disorder (OCD).^7-10^ Moreover, in OCD, compulsive behaviors driven by fear are associated with worse long-term outcomes.^11^

OCD can be found in about 4% of the general population.^12^ The disorder is characterized by changes in thinking (obsessions), changes in behavior (compulsions), or both. Obsessions are recurring, persistent, unwanted ideas, images or impulses, usually involving thoughts of harm, risk or danger, which cause anxiety and are intrusive. Compulsions (also known as rituals) are certain mental actions or acts that the person feels compelled to do in trying to reduce or avoid the anxiety caused by obsessions. Obsessions or compulsions are time-consuming (e.g., they take more than an hour a day) and they cause suffering and impairment in social, professional and other important areas of an individual’s life. In order to reduce the discomfort and suffering associated with these thoughts, patients can engage in maladaptive and exaggerated behaviors, also called compulsions or rituals. Among the most common OCD symptoms is the fear of contamination leading to excessive cleaning behaviors.^8,13,14^ Other frequent compensatory behaviors in OCD are the compulsion to wash hands, avoidance behavior to touch objects considered contaminated, and cleansing rituals. Avoidance of situations that can be considered as presenting a high risk of contamination can also occur, such as walking, using public transportation, sitting on a public park bench, or going to a public bathroom.^8,14^

Briefly, the cognitive-behavioral model of OCD proposes that undesirable intrusive thoughts activated by the most varied circumstances are part of normal cognitive activity. In most people, they disappear spontaneously; however, in individuals with OCD, these thoughts become obsessions due to the negative evaluations and interpretations associated with them. The evaluations and misinterpretations expressed in the form of catastrophic thoughts are responsible for emotional changes (fear, anxiety, guilt, anger). These feelings lead to the implementation of behaviors to ward off or eliminate the threat (through rituals, avoidance, neutralization) as well as to hypervigilance. After performing these behaviors, the temporary relief obtained reinforces the need to perform them again (negative reinforcement) as well as the distorted beliefs that underly and help perpetuate OCD symptoms.^15^

It has been described that patients with OCD, when in contact with objects considered contaminated, can feel the emergence of dysphoric symptoms, such as irritability, anxiety, and sadness.^8,16,17^ These symptoms have also been observed in people without mental disorders during the COVID-19 pandemic,^2,18,19^ and all these emotions and behaviors previously restricted to patients with OCD are now, in these unprecedented times, being considered the “new normal.” Therefore, the following question has been raised by psychologists and psychiatrists: What will be the psychological and behavioral impact of the pandemic in patients with obsessive-compulsive personality disorder or OCD, when their overestimated fantasies and fears become real concerns worldwide?

During the COVID-19 pandemic, the imminent risk of contamination creates a logical need for self-surveillance and hygiene habits, as recommended by the World Health Organization (WHO).^20^ From a different perspective, extreme hygiene measures have been encouraged by the media and by fake news, such as not touching objects and washing hands in a ritualized way. Other recommendations include the frequent use of hand sanitizer, cleaning with bleach any object that enters the home, removing “contaminated” shoes, having specific clothing to wear at home, and wearing gloves to touch objects because of the risk of contamination, among others. However, this kind of information can have drastic implications for individuals with OCD, since cognitive distortions and compensatory strategies (cleansing rituals) are no longer irrational or oversized, but rather become legitimate and socially accepted, generating plausible validation for the intensification of compulsive cleaning rituals.^16^

During infection pandemics, patients with OCD are prone to increase dysfunctional cleaning and organizing beliefs.^19^ Individuals with OCD are disturbed by excessive feelings of responsibility,^21,22^ as well as exaggerated and constant risk assessment – very common in patients with obsessions of contamination and compulsive washing behavior – and intolerance to uncertainty.^23^ In addition, the biased processing of information with an inclination to overestimate risk leads to a distorted evaluation of news related to COVID-19, which can hinder corrective experiences in the future, since probabilistic learning is impaired when dysfunctional beliefs are activated. This phenomenon was previously described in a study that found that patients with OCD had significantly lower levels of emotional control compared to healthy subjects when faced with an epidemic prediction task.^24^

Taken together, the double burden on the illness of these patients and the concern about a possible increase in the incidence of OCD during and after the pandemic period stand out. In a recent study conducted in China during COVID-19, high levels of OCD were observed.^19^ In addition, it is reasonable to predict that patients who presented remission of OCD symptoms would be more likely to have a relapse, and also that subclinical patients may scale up and ultimately be classified as having this disorder due to the reinforcement of their habits, emotions and thoughts. Finally, there is an increased chance of people developing other serious psychiatric disorders that can be disabling, such as mood and anxiety disorders.^16,17,25^ It is important to note that the intensification of obsessions, hopelessness, depressive symptoms and anxiety are associated with high rates of suicide in individuals with OCD.^26,27^ A systematic review and meta-analysis (with 48 studies) showed a median rate of suicidal ideation of 27.9% and suicide attempts of 10.3%.^26^ This is an important risk that needs to be considered and addressed by preventive strategies.

Regarding the treatment of OCD, there is a robust body of scientific evidence that corroborates the effectiveness of therapeutic approaches for this disorder, and many of them are freely available on the Internet through manuals, including self-applied techniques. The first-line psychological treatment proposed for OCD is cognitive-behavioral therapy (CBT), involving exposure and response prevention and including rituals and tolerance to uncertainty.^28^ Behavioral techniques favor exposure in order to prevent behaviors, mental responses or rituals that influence the perpetuation of OCD. However, such behaviors and responses are currently being stimulated by the media, particularly in the contamination and cleaning dimension. In this sense, online therapy has been shown as a promising therapeutic tool to develop cognitive behavior strategies with patients with OCD,^29,30^ and can be an alternative option to treat these patients in social distancing times. Finally, the authors suggest that combating fake news and disseminating honest, open data based on scientific and epidemiologic evidence is an obligation of social medias. In the same direction, good public health policies with adequate and balanced strategies could directly impact the control of OCD.

Therefore, in addition to the need to care for the mental health of the population, we emphasize that patients with OCD, especially those in the cleaning dimension, may be at a potentially higher risk during the current pandemic. To minimize possible damage, the dissemination of preventive and therapeutic psychoeducational materials for patients is suggested, as well as the provision of remote assistance channels. It is also essential that health professionals and health services like hospitals are trained to assess, diagnose and treat this portion of the psychiatrically vulnerable population.

Future investigations on the incidence and worsening of OCD during the pandemic and post-pandemic period are warranted, especially assessing the impact of this phenomenon on the quality of life of individuals, as well as on work and academic performance. Also, development of screening protocols in order to monitor OCD symptoms would be relevant to improve mental health policies and prevention or intervention programs to this population.
